# Expression of Murine 5-Aminolevulinate Synthase Variants Causes Protoporphyrin IX Accumulation and Light-Induced Mammalian Cell Death

**DOI:** 10.1371/journal.pone.0093078

**Published:** 2014-04-09

**Authors:** Erica J. Fratz, Gregory A. Hunter, Gloria C. Ferreira

**Affiliations:** 1 Department of Molecular Medicine, Morsani College of Medicine, Tampa, Florida, United States of America; 2 Department of Chemistry, University of South Florida, Tampa, Florida, United States of America; Boston University, United States of America

## Abstract

5-Aminolevulinate synthase (ALAS; EC 2.3.1.37) catalyzes the first committed step of heme biosynthesis in animals. The erythroid-specific ALAS isozyme (ALAS2) is negatively regulated by heme at the level of mitochondrial import and, in its mature form, certain mutations of the murine ALAS2 active site loop result in increased production of protoporphyrin IX (PPIX), the precursor for heme. Importantly, generation of PPIX is a crucial component in the widely used photodynamic therapies (PDT) of cancer and other dysplasias. ALAS2 variants that cause high levels of PPIX accumulation provide a new means of targeted, and potentially enhanced, photosensitization. In order to assess the prospective utility of ALAS2 variants in PPIX production for PDT, K562 human erythroleukemia cells and HeLa human cervical carcinoma cells were transfected with expression plasmids for ALAS2 variants with greater enzymatic activity than the wild-type enzyme. The levels of accumulated PPIX in ALAS2-expressing cells were analyzed using flow cytometry with fluorescence detection. Further, cells expressing ALAS2 variants were subjected to white light treatments (21–22 kLux) for 10 minutes after which cell viability was determined. Transfection of HeLa cells with expression plasmids for murine ALAS2 variants, specifically for those with mutated mitochondrial presequences and a mutation in the active site loop, caused significant cellular accumulation of PPIX, particularly in the membrane. Light treatments revealed that ALAS2 expression results in an increase in cell death in comparison to aminolevulinic acid (ALA) treatment producing a similar amount of PPIX. The delivery of stable and highly active ALAS2 variants has the potential to expand and improve upon current PDT regimes.

## Introduction

The first committed step of heme biosynthesis in non-plant eukaryotes and some prokaryotes, the pyridoxal 5′-phosphate (PLP)-dependent condensation of glycine and succinyl-coenzyme A to generate 5-aminolevulinate (ALA), coenzyme A (CoA), and CO_2_, is catalyzed by 5-aminolevulinate synthase (ALAS) [1], [2]. This reaction is directly coupled to the citric acid cycle via the substrate succinyl-CoA and is the key regulatory step of heme biosynthesis [3]. In mammals, two chromosomally distinct genes each encode an ALAS isoenzyme, and the two isoenzymes are differentially expressed in a tissue specific manner [4]. The human gene for the non-specific or housekeeping isoform, ALAS1, is located on chromosome 3 [5], [6], and is expressed ubiquitously in all tissues [7]. The gene encoding the erythroid specific isoform, ALAS2, is located on the x-chromosome [6], [8] and is expressed only in developing erythroblasts [7].

The two ALAS isoenzymes are translated as precursor proteins with N-terminal mitochondrial matrix import signal sequences that are proteolytically cleaved following importation to yield the mature enzymes [9]–[12]. The activity of the enzyme is only manifested upon localization to the mitochondrial matrix, as this is where the substrate succinyl-CoA is produced [13]–[16]. An important aspect of the import sequences in both ALAS1 [17] and ALAS2 [18] are the presence of heme-regulatory motifs (HRMs), which consist of short amino acid sequences characterized in part by adjacent cysteine-proline (CP) residues [19]. HRMs confer heme-binding properties and have been shown to function as heme-oxygen sensors in bacteria [20], yeast [21] and mammals [18], [22], [23]. In *in vitro* translocation experiments with isolated mitochondria and ALAS2 precursor protein, the two heme-binding motifs in the leader sequence, corresponding to C11 and C38 in murine ALAS2 (mALAS2), were reported to bind heme and prevent translocation of precursor ALAS2 into the mitochondrion [18]. Structural and biochemical data have also demonstrated that a heme-peptide interaction occurs between hemin and the presequence of ALAS2 [24], further indicating the potential of heme to act as a feedback inhibitor of the pathway by preventing the mitochondrial import of precursor ALAS2 when heme levels are sufficient for cellular requirements.

Much of what we know about the chemical and kinetic mechanisms of ALAS2 comes from *in vitro* enzymatic assays that have helped establish and define the microscopic steps of the ALAS-catalyzed reaction, including the rates of glycine and succinyl-CoA binding, formation of the quinonoid intermediates, and product release [2], [25]–[28]. These studies, performed using mALAS2 purified from *E. coli* cells expressing the recombinant mature enzyme, have led to an understanding of the importance of specific regions and single amino acid residues in the intrinsic activity of ALAS2 [29]–[35]. Generally, a mutation made to an amino acid predicted to be of functional importance causes a decrease in activity of the enzyme. For example, K313 of mALAS2 was identified as the amino acid involved in the Schiff base linkage with the PLP cofactor [36], and mutations in K313 completely abolish measurable activity of mALAS2 under standard assay conditions [29], [37]. However, some mutations in mALAS2 cause significantly increased activity of the enzyme, as demonstrated both using purified enzyme [31], [35], and in bacteria when expressing plasmids encoding the variant enzymes [35]. These mutations are all located in an extended conformation region termed the active site loop (Y422-R439), which is predicted to act as a “lid” over the active site following substrate binding [35]. Thus, ALAS is thought to undergo a conformational change from an open conformation, in which the substrates glycine and succinyl-CoA can bind in the active site, to a closed conformation, during which the products ALA, CoA, and CO_2_ are formed [25], [28]. When the reaction is complete, the active site loop reopens, and the products are released. It is the opening of the active site loop to allow product release that limits the overall rate of the enzymatic reaction [2], [25], [27]. Based on a combination of kinetic [25], [28] and structural modeling studies [38], it was proposed that mutations in the active site loop can lead to hyperactive forms of ALAS, defined as those with at least a 10-fold increase in catalytic efficiency toward one or both substrates, by accelerating reversion to the open loop conformation upon product formation [35].

Since ALAS catalyzes the rate-determining step of tetrapyrrole biosynthesis in mammals [2], [25], overexpression of ALAS in prokaryotic [35] and eukaryotic cells [39] results in accumulation of the photosensitizing heme precursor, protoporphyrin IX (PPIX). This property has potential for applications of ALAS or ALAS variants in photodynamic therapy of tumors and other non-malignant dermatological indications, such as acne vulgaris, psoriasis, and scleroderma [40], [41]. In this study, we transfected mammalian cells with mALAS2 variants and measured PPIX accumulation using fluorescence activated cell sorting (FACS). We identified the R433K variant with additional mutations of the HRMs in the presequence as the variant causing the most cellular PPIX accumulation. Subsequently, we used the variants causing the most PPIX accumulation to study the cell death caused by the PPIX toxicity and photosensitization.

## Materials and Methods

### Materials

5-Aminolevulinic acid hydrochloride (ALA) was purchased from Acros Organics (Morris Plains, NJ), and dissolved in distilled water at a concentration of 10 mg/mL. Glycine, purchased from Fisher Chemical (Fairlawn, NJ), was dissolved in phenol red-free culture medium purchased from Mediatech, Inc. (Manassas, VA) to give a stock concentration of 1 M. Paclitaxel (6 mg/mL stock in DMSO) was graciously provided by the laboratory of Dr. Scott Antonia (H. Lee Moffitt Cancer Center and Research Institute, Tampa, FL), and was diluted in culture medium directly before use. Deferoxamine mesylate, purchased from Sigma (St. Louis, MO), was dissolved in distilled water to create a 10 mM stock solution. Propidium iodide, 4′,6-diamidino-2-phenylindole (DAPI) and kanamycin sulfate were purchased from Acros Organics (Morris Plains, NJ), and 3-(4,5-dimethylthiazol-2-yl)-2,5-diphenyltetrazolium bromide (MTT) was purchased from Sigma-Aldrich (St. Louis, MO). BmtI and BamHI restriction enzymes were obtained from New England BioLabs, Inc. G418 sulfate was procured from Mediatech, Inc. (Manassas, VA).

### Plasmids

The precursor ALAS2 cDNAs were individually subcloned into the multiple cloning site of the pIRES2-ZsGreen1 vector (purchased from Clontech Laboratories, Inc. Mountain View, CA) using the BmtI and BamHI restriction sites. Digested ALAS2-encoding fragments were ligated into the digested pIRES2-ZsGreen1 vector using T4 DNA ligase and ligase buffer (Thermo Scientific Fermentas (Waltham, MA)). Electrocompetent BL21(DE3) cells were transformed by electroporation with the ligated plasmid DNA and selected by spreading them on LB agar medium containing 10 μg/mL kanamycin sulfate. Plasmid DNA was purified from a single colony using a QiaPrep Spin Miniprep kit (Qiagen Inc.; Germantown, MD), and the sequence of the cloned DNA was verified by Genewiz, Inc. (New Brunswick, NJ).

### Cell Culture

K562 human immortalized myelogenous leukemia cells (ATCC) were maintained in RPMI-1640 culture medium, purchased from Mediatech, Inc. (Manassas, VA), with 10% fetal bovine serum (FBS; purchased from Thermo Scientific Waltham, MA), gentamicin (50 μg/mL), penicillin (60 μg/mL) and streptomycin (100 μg/mL) at 37°C in a humidified incubator with 5% CO_2_. HeLa human cervical carcinoma cells (ATCC) were maintained in DMEM culture medium with 4.5 g/L glucose, L-glutamine, and sodium pyruvate (Mediatech, Inc.; Manassas, VA), with 10% fetal bovine serum (FBS), gentamicin (50 μg/mL), penicillin (60 μg/mL) and streptomycin (100 μg/mL) at 37°C in a humidified incubator with 5% CO_2_.

### Stable Transfection of K562 Human Erythroleukemia Cells

K562 cells were transfected with Lipofectamine LTX and PLUS Reagent, purchased from Invitrogen (San Jose, CA), according to the supplier’s optimized protocol for K562 cells. On the day of transfection, a hemocytometer and trypan blue staining were used to count the cells and determine culture density and viability. In a 6-well plate, K562 cells (5×10^5^ cells per well) were seeded in a 6-well plate at a volume of 2 mL of RPMI-1640 growth medium with 10% FBS 30 minutes prior to transfection. For each transfection, 2.5 μg of DNA was added into 500 μL of RPMI medium without serum. 2.5 μL of PLUS reagent (at a 1∶1 ratio to DNA) was then added directly to the diluted DNA. After gentle mixing and a 10 minute incubation at room temperature, 10 μL of Lipofectamine LTX was added into the diluted DNA solution, mixed gently and incubated for 35 minutes at room temperature to form DNA-Lipofectamine LTX complexes. The DNA-Lipofectamine LTX complexes were added dropwise to each well containing cells and mixed gently by manually rocking the plate back and forth. 24 hours after transfection, the cells were pelleted by centrifugation at 400×g and resuspended in RPMI-1640 culture medium with 10% FBS, gentamicin (50 μg/mL) and the selection antibiotic, G418 sulfate (500 μg/mL). Every 2–3 days, the cells were pelleted and resuspended in fresh medium with 10% FBS, gentamicin (50 μg/mL), and G418 sulfate (500 μg/mL). Assays were performed 2–6 weeks after the start of selection.

### Transient Transfection of Hela Cells

On the day prior to transfection, HeLa cells were trypsinized and counted. Approximately 2×10^4^ cells were seeded into each well of a 24-well plate in 0.5 mL of DMEM. Cell density was ∼30–50% confluent on the day of transfection. For each transfection, 250 ng of DNA was diluted into 100 μl of DMEM without serum. 1 μl of Lipofectamine LTX was added into the diluted DNA solution, mixed gently and incubated for 30–45 minutes at room temperature to form DNA-Lipofectamine LTX complexes. The DNA-Lipofectamine LTX complexes were added dropwise to each well containing cells and mixed gently by manually rocking the plate back and forth for a few seconds. After 4 hours of incubation with the DNA-Lipofectamine LTX complexes, the medium was aspirated out of each well and fresh DMEM with 10% FBS, gentamicin (50 μg/mL), penicillin (60 μg/mL) and streptomycin (100 μg/mL) was added to each well of cells. Cells were incubated at 37°C in a CO_2_ incubator for 24 or 48 hours post-transfection before assaying.

### Preparation of Cells for FACS and Quantitation of PPIX

While K562 cells were suspended in phenol red-free medium, HeLa cells were washed, scraped and resuspended in phosphate-buffered saline (PBS; 80 mM disodium hydrogen orthophosphate, 20 mM sodium dihydrogen orthophosphate, 100 mM sodium chloride, pH 7.5) before pipetting into BD Falcon tubes with cell strainer caps. Preparation of either K562 or HeLa cells for FACS was done under very low light conditions (1–2 Lux as measured by a Pyle PLMT68 light meter) in order to minimize phototoxicity caused by PPIX accumulation. FACS analyses were performed using a BD LSR II Analyzer (Becton, Dickinson, and Company) and FACSDiva Version 6.1.3 software. ZsGreen1 emission was measured between 515 nm and 545 nm (530/30BP filter) when cells were excited using the 488 nm laser. In order to eliminate any background red fluorescence, the 633 nm-red laser was blocked during the collection of the PPIX emission data. PPIX emission was determined in the 619 nm and 641 nm range (630/22BP filter) when cells were excited with the 405 nm laser. Forward-scatter (FSC) *versus* side-scatter (SSC) dot plots were used to gate the whole cells and thus remove the contribution of the cell debris from the population being examined. A minimum of 10,000 of the gated whole cells were then depicted in dot plots of SSC *versus* ZsGreen1 fluorescence, and the “green-fluorescent population” gate was defined based on untransfected HeLa cells as negative controls. Dot plots of SSC *versus* PPIX fluorescence were used to define the PPIX-accumulating cells for both the “green-fluorescent” and the “non-green fluorescent” populations. The PPIX gating was based on the negative control for PPIX, the pIRES2-ZsGreen1 vector-expressing cells. PPIX fluorescence values were normalized for transfection efficiency using the corresponding ZsGreen1 fluorescence value.

### Light Exposure Assays

HeLa cells in 24-well plates were transfected 48 hours prior to FACS analysis. Cells were washed twice with PBS and placed on ice (to prevent overheating) underneath a Sylvania incandescent flood lamp (150W, 120V) for 10 minutes. Light intensity at the samples was measured before each light exposure experiment using a Pyle PLMT68 light meter, and it was between 21–22 kLux for all experiments. The samples were resuspended and pipetted into tubes with cell strainer caps and analyzed first for ZsGreen1 and PPIX fluorescence (as described above), and then for cell viability. In order to determine the statistical significance of cell death directly attributable to mALAS2-induced PPIX accumulation and phototoxicity, samples were compared to the ZsGreen1 controls during t-test evaluations.

### Cell Viability Assays

Cell death was assessed by measuring incorporation of either propidium iodide or DAPI into nuclear DNA, as these fluorescent dyes cross the plasma membrane in dying cells much more efficiently than in live cells. HeLa cell cultures were independently incubated with the fluorescent DNA-binding dyes propidium iodide (10 μg/mL) and DAPI (10 μg/mL) for 5 min at 22°C. The cells were then analyzed by determining the fluorescence intensities using FACS. Fluorescence emission of nucleic acid-bound propidium iodide was measured between 585 nm and 625 nm (605/40BP filter) upon excitation of the cells using the 488 nm laser, while DNA-bound DAPI emission was measured between 440 nm and 460 nm (450/20BP filter) following excitation of the cells at 375 nm. Dot plots of SSC *versus* propidium iodide or DAPI fluorescence were used to define the respective gates, which were based on the control samples in which no propidium iodide or DAPI were added. Cell viability was independently determined using the MTT dye reduction assay as described elsewhere [42], [43]. HeLa cells (5×10^3^ cells per well) were seeded in 96-well plates, and on the following day were transfected with expression plasmids for mALAS2 variants as described above. Twenty-four hours after plasmid transfection, or 4 hours after addition of 100 μM ALA, cells were exposed to light for 10 minutes. The culture medium was pipetted out, 50 μL of 2 mg/mL MTT was added to each well, and cells were incubated for an additional 4 hours at 37°C. Upon solubilization of the cells with DMSO (100 μL/well) during a 10 minute incubation, the solubilized, MTT-treated cells were thoroughly mixed by pipetting several times, and absorbance was measured at 540 nm using a μQuant plate reader (Bio-Tek Instruments, Inc.).

### Confocal Fluorescence Microscopy

HeLa cells were grown in Thermoscientific Nunc Labtek sterile 4-well chambered coverglass until 50% confluent, and then transfected as described above. Six hours later, the culture medium was supplemented with glycine to yield a final concentration of 100 mM, and immediately before obtaining confocal fluorescence microscopy images of the cells, the medium was removed from the wells and the cells were washed with PBS three times. Live cell imaging was performed using a 3i-Olympus spinning disk confocal microscope operated by Slidebook 5 software and equipped with a Photometrics Evolve EMCCD camera. The filter block used consisted of a 350/50 nm excitation filter, a BS400 beamsplitter, and a 630/75 nm emission filter.

## Results

### Transient Expression of Malas2 Variants Causes Accumulation of PPIX in HeLa Cells

**Figure 1:**
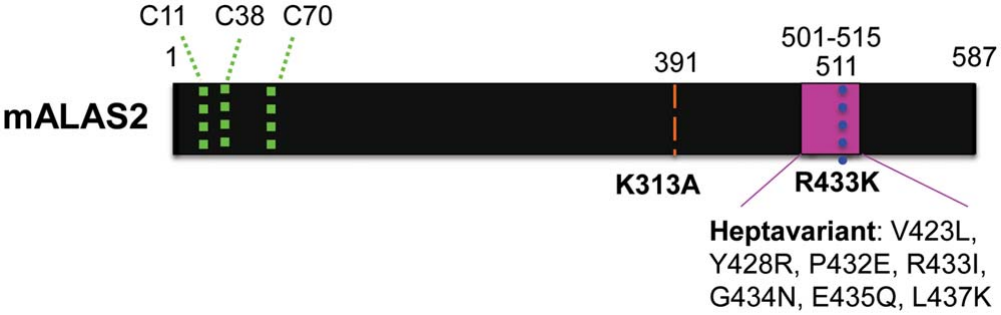
Murine ALAS2 protein schematic indicating mutated amino acid residues. The green dotted lines represent the relative locations of the three cysteines in the HRMs of ALAS2. C11 and C38 are in the ALAS2 presequence. K313A, R433K, and the heptavariant mutations and their respective positions are indicated according to the amino acid numbering previously described for mature mALAS2 [31], [35], [36]. The amino acid positions according to the numbering for the precursor enzyme are written above the diagram.

**Figure 2:**
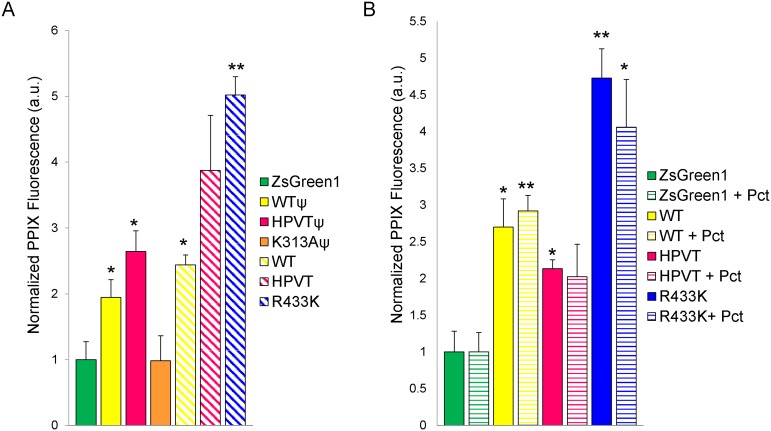
Porphyrin accumulation in transiently transfected HeLa cells with mALAS2 WT- and variant-expressing plasmids. (A) HeLa cells transfected with mALAS2 variants with and without (^Ψ^) mutated presequences analyzed 24 hours after transfection. (B) HeLa cells transfected with mALAS2 variants with mutated presequences, in cells cultured in the absence or presence of 10 nM paclitaxel (Pct), analyzed 48 hours after transfection. Normalized PPIX fluorescence values were obtained by first dividing the mean PPIX fluorescence by the mean ZsGreen1 fluorescence for each cell population and secondly dividing by the mean PPIX fluorescence/ZsGreen1 fluorescence ratio of the pIRES2-ZsGreen1-transfected cells. Each data set represents three separate experiments ± standard deviation (*p<0.05 and **p<0.01, Student’s *t*-test) [a.u., arbitrary units].

HeLa cells were transfected with mALAS2-encoding plasmids with or without mutations in the mALAS2 presequence and/or mature enzyme sequence (Table 1, Figure 1). Where indicated, the mALAS2 presequence was mutated at three cysteine residues, C11, C38, and C70, in order to yield nonfunctional HRMs and thus eliminate heme feedback inhibition of mitochondrial import [18], [40]. The plasmids were designed such that a single bicistronic mRNA, encoding both mALAS2 and the fluorescent protein ZsGreen1, separated by an internal ribosomal entry site (IRES), would be produced. Transcriptional expression of mALAS2 and ZsGreen1 was under control of the constitutively active cytomegalovirus (CMV) promoter [44]. Twenty-four hours post-transfection, wild-type mALAS2 with a wild-type presequence (WT^Ψ^) expression caused a slight, but statistically significant as defined by Student’s t-test (p<0.05), increase in PPIX accumulation (Figure 2A). The K313A mutation in ALAS2 leads to undetectable enzymatic activity values *in vitro* [45], and under *ex vivo* conditions this also appears to be the case, as no PPIX accumulated in K313A-expressing cells (K313A^Ψ^), a result similar to that of the mammalian cells harboring the pIRES2-ZsGreen1 vector (ZsGreen1) alone (Figure 2A). In comparison to WT^Ψ^, the heptavariant mutations in the active site loop alone (HPVT^Ψ^) and mutations in the presequence alone (WT) caused a statistically significant (p<0.05) increase in PPIX production in expressing cells (Figure 2A). The combination of the heptavariant mutations in the active site loop and mutations in the presequence (HPVT) resulted in variable PPIX production that was not statistically significant. Somewhat unexpectedly, it was expression of the mALAS2 variant with a mutated presequence containing the R433K mutation (R433K) that caused the largest accumulation of PPIX. Mutation of R433 residue to a lysine in the purified, mature enzyme results in an increase in activity to twice that of the wild-type enzyme (*i.e*., a 2-fold increase in the *k*_cat_ value and a 1.65 to 1.85-fold enhancement in the specificity constants for glycine and succinyl-CoA over those of wild type, mature mALAS2) [31]. Similarly, the increase in PPIX accumulation in HeLa cells expressing the R433K precursor with a mutated presequence was approximately 2.5-fold compared to the cells expressing WT^Ψ^ and 2-fold in comparison to cells expressing WT (Figure 2A).

**Table 1:** Plasmid constructs used to express murine ALAS2 variants in mammalian cell lines.

Plasmid Name	Mutations in	
	mALAS2 Presequence	Mature mALAS2 Sequence
pIRES2-ZsGreen1	N/A^1^	N/A^1^
pEF21	---^2^	---^2^
pEF22	---^2^	V423L,Y428R,P432E, R433I, G434N, E435Q and L437K
pEF23	---^2^	K313A
pEF24	C11S, C38S and C70S	---^2^
pEF25	C11S, C38S and C70S	V423L,Y428R,P432E, R433I, G434N, E435Q and L437K
pEF26	C11S, C38S and C70S	K313A
pEF31	C11S, C38S and C70S	R433K

1 N/A, non-applicable;

2 ---, no mutations.

Because PPIX can be used as a photosensitizing agent for PDT of cancers, we examined the effect of combining mALAS2-induced PPIX accumulation with the chemotherapeutic drug paclitaxel, a well-characterized inhibitor of mitosis [46]–[48]. Only mALAS2 variants with mutated presequences were used in the drug combination experiments, since those variants presented the most potential for PPIX accumulation, and thus, subsequent cell death resulting from light-induced phototoxicity. A dose response curve revealed that the concentration of paclitaxel that killed 25% of HeLa cells in 48 hours (IC25) was 10 nM (data not shown), and this concentration was chosen as the desired concentration for the PDT combination experiments. Paclitaxel had no statistically significant effect, as defined by Student’s t-tests, on the amount of accumulated PPIX in the mammalian cells transfected with any of the ALAS2 variants after 48 hours (Figure 2B). The largest increase in PPIX fluorescence was observed with the R433K variant, regardless of whether or not paclitaxel was included in the culture medium (Figure 2B).

### Supplementation of Cell Culture Medium with Glycine Leads to Increased PPIX Accumulation in Malas2-expressing HeLa Cells

**Figure 3:**
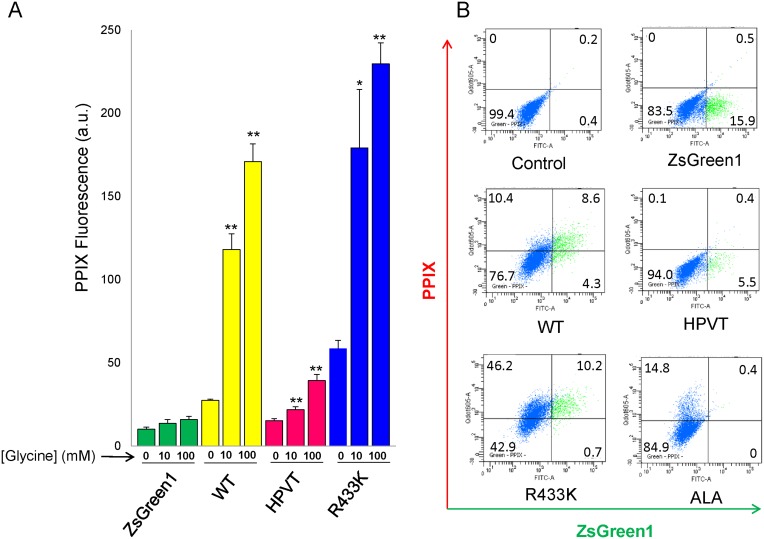
Glycine supplementation of culture medium increases PPIX accumulation in HeLa cells expressing murine wild-type, HPVT, and R433K ALAS2. (A) PPIX fluorescence measured by flow cytometry 24 hours after transfection and 18 hours after supplementing culture medium with 10 mM glycine or 100 mM glycine. The culture medium of the corresponding control cells and control transfected cells was not supplemented with glycine. Normalized PPIX fluorescence values were obtained by dividing the mean PPIX fluorescence by the mean ZsGreen1 fluorescence for each cell population. Mean PPIX fluorescence values are representative of three separate experiments ± standard deviation (*p<0.05 and **p<0.01, Student’s *t*-test) [a.u., arbitrary units]. (B) ZsGreen1 fluorescence *versus* PPIX fluorescence in HeLa cell populations supplemented with 100 mM glycine as measured by flow cytometry. In each dot plot, cells in the green-fluorescent range are represented in green and to the right of the vertical black line, while cells that are not green-fluorescent are in blue. Cells that are red-fluorescent due to PPIX accumulation are above the horizontal black line, while cells that are not red-fluorescent are below the horizontal black line. The percentage of cells in each quadrant is written in the corners of each dot plot. “Control” refers to non-transfected HeLa cells.

**Figure 4:**
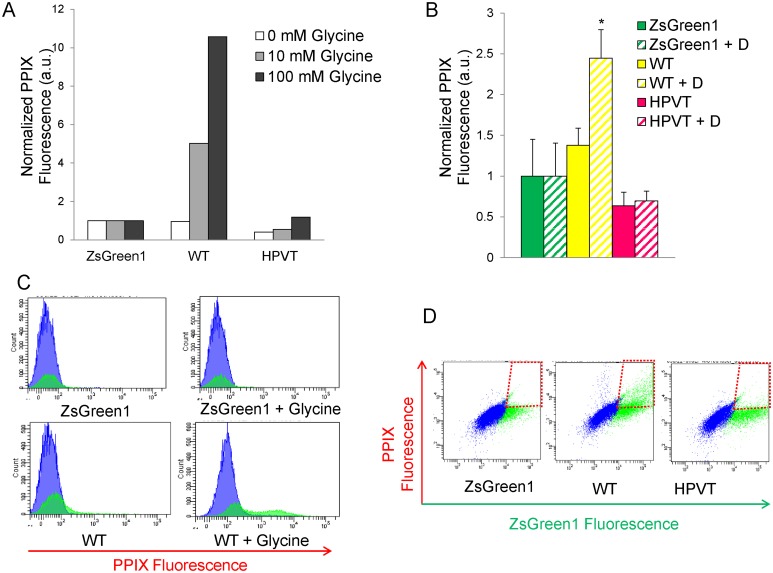
Stable expression of murine ALAS2 variants in K562 cells results in PPIX accumulation when culture medium is supplemented with either glycine or deferoxamine. (A) PPIX fluorescence 18 hours after supplementation of the culture medium with glycine to yield 10 mM and 100 mM final concentrations. Normalized fluorescence values were obtained by first dividing the mean PPIX fluorescence by the mean ZsGreen1 fluorescence for each cell population and secondly dividing by the mean PPIX fluorescence/ZsGreen1 fluorescence ratio of the pIRES2-ZsGreen1-transfected cells. (B) PPIX fluorescence 18 hours after supplementation of the culture medium with deferoxamine to a final concentration of 100 μM (+D). (C) PPIX fluorescence of K562 cell populations supplemented with glycine to a 100 mM final concentration. The blue area represents cells that do not express ZsGreen1, and the green area represents the population that expresses ZsGreen1. K562 cells that express ZsGreen1 are not affected by glycine supplementation of the culture medium (upper plots), while cells that express WT exhibit a significant increase in PPIX fluorescence when the culture medium is supplemented with glycine (lower plots). (D) PPIX fluorescence of K562 cell populations grown in culture media containing 100 μM deferoxamine. Cell growth in the presence of deferoxamine significantly increases the PPIX fluorescence in K562 cells expressing WT, but does not affect PPIX fluorescence in cells expressing only ZsGreen1 or HPVT, as indicated by the number of cells within the red dotted-line boxes.

Given that PPIX is a photosensitizer [49] and the Michaelis-Menten constant (*K*_m_) of mALAS2 for glycine, at 25±4 mM [3], [25], [34], is significantly higher than its intracellular concentration of approximately 2.5 mM [50], it is plausible that supplementation of the cell culture medium with glycine would lead to enhanced synthesis of ALA, and consequently, PPIX, which in turn might increase the efficacy of PPIX-induced phototoxicity. To examine whether increased glycine concentration caused enhanced PPIX accumulation, HeLa cells transfected with mALAS2 (wild type and variants) were grown in medium with different glycine concentrations (Figure 3A). Samples with culture medium supplemented with 100 μM ALA for 4 hours served as a control for PPIX accumulation independent of glycine concentration (Figure 3B). The concentration and treatment times for ALA were chosen based on experiments indicating that the extent of PPIX accumulated in HeLa cells supplemented with 100 μM ALA was similar to that of HeLa cells expressing R433K, the mALAS2 variant that induces the highest levels of PPIX accumulation in these cells (Figure S1). HeLa cells expressing WT, HPVT or R433K in culture medium supplemented for 18 hours with 10 mM or 100 mM glycine exhibited significant increases in PPIX in comparison to the “no glycine” controls, with R433K again demonstrating the highest PPIX accumulation (p<0.01) (Figure 4A). Supplementing the culture medium with either 10 mM or 100 mM glycine for cells expressing WT resulted in approximately 4- and 6-fold increases in PPIX accumulation, respectively, as compared to no glycine supplementation. For cells expressing R433K, glycine supplementation more than tripled the PPIX accumulation, representing a fourteen-fold increase over the control HeLa cells supplemented with glycine. Cells expressing HPVT were also affected by addition of either 10 mM or 100 mM glycine, increasing the PPIX by 1.4-fold and 2.5-fold, respectively. Glycine elicited no effect on PPIX production in HeLa cells alone, those treated with 100 μM ALA, or HeLa cells expressing the pIRES2-ZsGreen1 vector (Figure 3B).

### Glycine and Deferoxamine Increase PPIX Accumulation in Malas2-expressing K562 Cells

We generated stable K562 human myelogenous erythroleukemia cell lines expressing mALAS2, expecting that these cells might accumulate high amounts of PPIX, due to their similarity to undifferentiated erythrocytes [51]. We expressed only WT and HPVT in order to test if HPVT could have higher activity in a cell line in which ALAS2 in normally expressed. Initially, the stable cell lines expressing WT did not show an increase in PPIX fluorescence as compared to regular K562 cells (Figure 4A), and we postulated that glycine availability was again a limiting factor, as we had observed in HeLa cells (Figure 3). Another possible limitation upon PPIX accumulation could be a more effective conversion of PPIX into heme in K562 cells, in which case the inclusion of an iron-specific chelator such as deferoxamine should increase the PPIX fluorescence by reducing or even preventing this conversion. To address why the expression of mALAS2 alone did not cause a larger increase in PPIX and what could limit the ALAS2-induced PPIX accumulation, the culture medium of K562 cells expressing (1) ZsGreen1 control, (2) WT, or (3) HPVT was supplemented with glycine to yield final concentrations of 10 mM or 100 mM glycine (Figure 4A and 4C) or deferoxamine mesylate to yield a final concentration of 100 μM deferoxamine mesylate (Figure 4B and 4D). Expression of WT in K562 cells did not increase the cellular PPIX fluorescence, but PPIX fluorescence increased by more than 5- and 10-fold when the culture medium contained 10 mM glycine and 100 mM glycine, respectively (Figure 4A). No significant differences were seen in cells not expressing mALAS2 (Figure 4C) or in cells expressing HPVT when treated with 10 mM glycine (Figure 4A). However, in contrast to HeLa cells, when 100 mM glycine was used, cells expressing HPVT did exhibit an increase in PPIX fluorescence by approximately 3-fold (Figure 4A).

Deferoxamine is a well-characterized iron-specific bacterial siderophore with a long history of clinical use in iron chelation therapy. Deferoxamine has the potential to increase PPIX by decreasing the cellular iron concentration, thereby inhibiting the conversion of PPIX to heme [52], [53]. Treatment of K562 cells with deferoxamine for 18 hours caused no change in PPIX fluorescence in cells not expressing mALAS2 or cells expressing HPVT. Deferoxamine did cause a significant increase in PPIX in cells expressing WT, in which case the mean PPIX fluorescence increased from 1.4-fold over cells expressing ZsGreen1, to 2.4-fold with deferoxamine (Figure 4B and 4D).

### PPIX Primarily Accumulates and Localizes at the Plasma Membrane in HeLa Cells Expressing R433K

**Figure 5:**
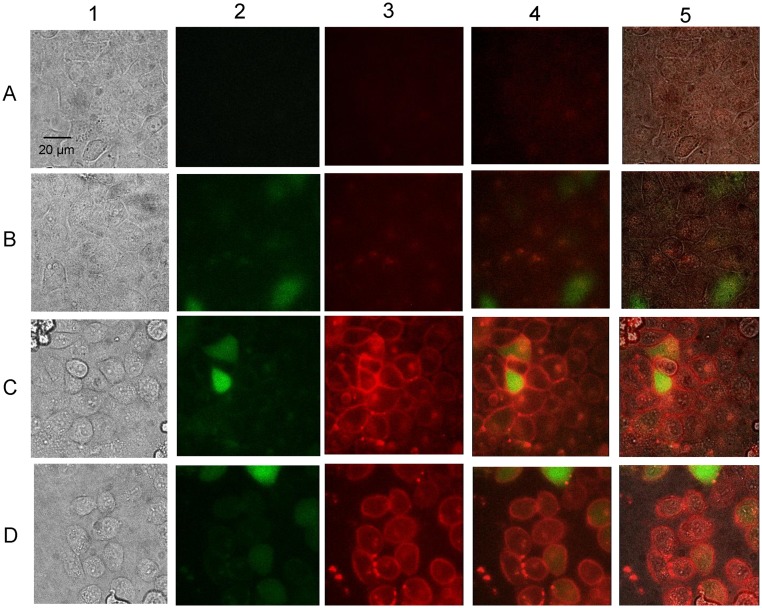
Fluorescence microscopy of HeLa cells expressing GFP and the R433K mALAS2 variant. From left to right for each row, the panels correspond to (1) brightfield, (2) green fluorescence, (3) red fluorescence, (4) superimposed green and red fluorescence, and (5) superimposed brightfield, green fluorescence, and red fluorescence images. (A) HeLa cells grown to confluence in culture medium with 100 mM glycine. (B) HeLa cells transfected with pIRES2-ZsGreen1 and grown to confluence in culture medium with 100 mM glycine. (C) HeLa cells transfected with pEF31, which expresses the R433K variant from the CMV promoter in the pIRES2-ZsGreen1 vector (Table 1), and grown to confluence in culture medium with 100 mM glycine. (D) HeLa cells transfected with pEF31 and grown to subconfluence (*i.e.,* 80% confluence) in culture medium with 100 mM glycine. Pictures were obtained 24 hours after transfection and 18 hours after glycine supplementation. ZsGreen1 (green) is present throughout the cell, while PPIX (red) accumulates at the plasma membrane of cells expressing R433K and surrounding cells.

To evaluate cellular intactness and accumulated PPIX distribution in the transfected HeLa cells, we used confocal microscopy to visualize the fluorescent PPIX in individual HeLa cells (Figure 5A) and HeLa cells transfected with either the pIRES2-ZsGreen1 vector (Figure 5B) or pEF31 (harboring R433K) (Figures 5C and 5D). Transfected cells were grown in medium supplemented with 100 mM glycine for 18 hours in preparation for imaging. The outline of intact cells, and thus their morphological integrity, was evident in the three cases. As expected, green fluorescence was visualized in the transfected (Figures 5B, columns 2, 4 and 5, 5C, columns 2, 4 and 5 and 5D, columns 2, 4 and 5) but not control HeLa cells (Figure 5A, columns 2, 4 and 5). In fact, green fluorescence, arising from the soluble ZsGreen1 green fluorescent protein was observed evenly distributed throughout the cytoplasm of the HeLa cells transfected with either pIRES2-ZsGreen1 (Figure 5B) or the R433K-expression plasmid (Figures 5C and 5D). While the enzyme protoporphyrinogen oxidase catalyzes PPIX production exclusively within the mitochondrion, PPIX accumulated primarily within the plasma membranes of HeLa cells expressing R433K, as visualized by the characteristic red fluorescence; however, PPIX build-up was also apparent within the cells (Figures 5C, columns 3–5 and 5D, columns 3–5). Thus, it appears that much of the PPIX produced in the mitochondria eventually accumulates in the plasma membrane, entirely consistent with the fact that PPIX is a relatively lipophilic molecule. Of note, PPIX accumulated in not only transfected cells, but also in surrounding cells, indicating that PPIX could leave the transfected cells and be taken up by nearby cells (Figures 5, columns 3–5 and 5D, columns 3–5C). It is very likely that, additionally, PPIX accumulated in organelle membranes within the cell, such as in the mitochondrion, but since PPIX photobleached within a few seconds under the conditions utilized here, it was not possible to obtain high-resolution organelle images with our current microscopic parameters. Finally, the cobblestone-like morphology, instead of a slightly elongated shape, of the HeLa cells may result from the cell density and/or the nature of the growth surface. HeLa cells tend to adopt a more cobblestone-like appearance as cultures approach confluence and the HeLa cell morphology varies with the adhesion surface.

### ALAS2-induced PPIX Accumulation Followed by Light Exposure Combined with Paclitaxel Treatment Causes Cell Death

**Figure 6:**
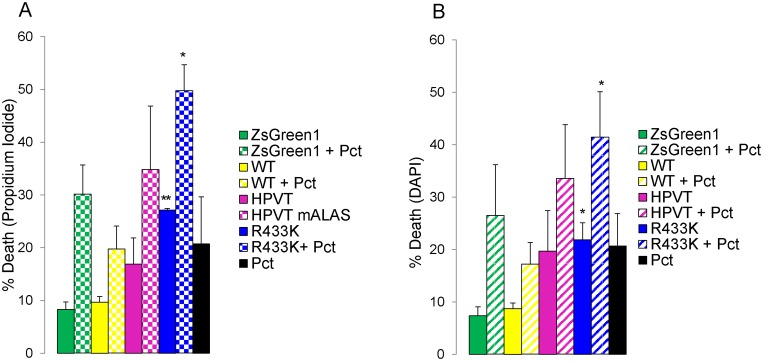
Light-induced cell death of HeLa cells expressing mALAS2 variants. (A) Cell death measured by changes in DNA-bound propidium iodide fluorescence. (B) Cell death measured by changes in DNA-bound DAPI fluorescence. HeLa cells were transfected with expression plasmids for either wild type (WT) or mALAS2 variants and treated with 10 nM paclitaxel 4-hour post-transfection. 48 hours after transfection, cells were stained and analyzed by flow cytometry. Cell death percentage values were compared to that of the pIRES2-ZsGreen1-transfected cells control. Each data set represents three separate experiments ± standard deviation (*p<0.05 and **p<0.01, Student’s *t*-test).

Both propidium iodide and DAPI staining were utilized to assess cell viability after mALAS2-induced PPIX accumulation, light exposure, and paclitaxel treatment, based on the specific fluorescence emission of each dye when bound to the DNA of intact cells. Stains were added to cell samples 48 hours after transfection, and the fluorescence of DNA-bound propidium iodide and DAPI were measured by flow cytometric analysis using the respective fluorescence emission maxima of 613 nm and 460 nm. Expression of WT or HPVT did not significantly increase cell death following light exposure. However, expression of R433K caused a statistically significant (p<0.05) increase in cell death of up to 30%, as measured both by propidium iodine (Figure 6A) and DAPI (Figure 6B) staining, in comparison to expression of ZsGreen1 alone. Addition of paclitaxel increased cell death in all samples, including controls, by 10–25%. HeLa cells expressing R433K and treated with paclitaxel exhibited the highest percentage of up to 50% cell death (Figure 6A–B). Combination of paclitaxel with mALAS2-induced PPIX accumulation and light treatments exhibited an additive effect in causing death in HeLa cells.

### Supplementation of Cell Culture Medium with Glycine Enhances Phototoxicity and Cell Death in Malas2-expressing HeLa Cells

**Figure 7:**
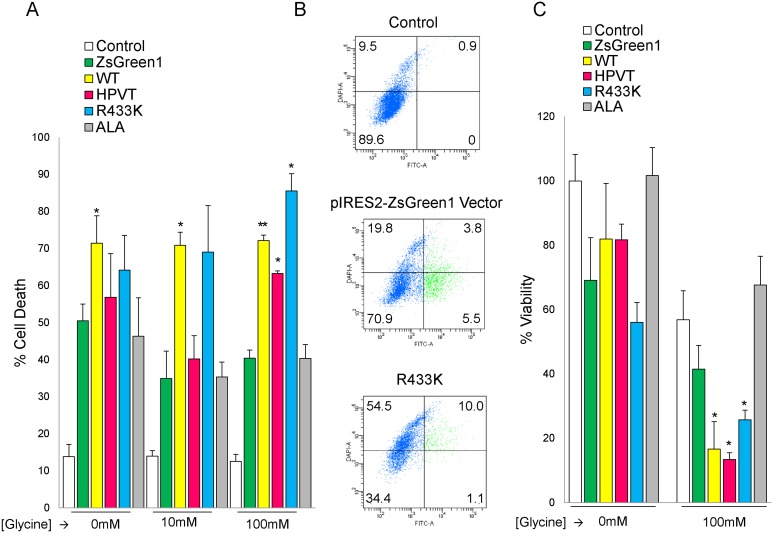
Light-induced cell death in murine ALAS2 variants-expressing HeLa cells cultured in a medium supplemented with glycine. (A) Cell death as assessed by DAPI fluorescence. Six hours after transfection of the HeLa cells with the different expression plasmids, the cells were grown for an additional 18 hours in medium without or with glycine (10 mM or 100 mM). Cell death percentage values are representative of three separate experiments ± standard deviation (*p<0.05, Student’s *t*-test). For control and ALA-treated cells, cell death was calculated by dividing the number of DAPI-stained cells by the total number of cells. For ZsGreen1-, WT-, HPVT-, and R433K-expressing cells, cell death was calculated from the ZsGreen1-positive cell populations by dividing the number of DAPI-stained cells by the total number of ZsGreen1-positive cells. (B) Cell death in HeLa cell populations grown in culture medium supplemented with glycine to a final concentration of 100 mM. The percentage of cells in each quadrant is written in the corners of each dot plot. (C) Cell viability as measured by metabolism of 3-(4,5-dimethylthiazol-2-yl)-2,5-diphenyltetrazolium bromide (MTT). Cells were transfected 24 hours before light treatment, and 100 mM glycine was added 18 hours before light treatment. Cell viability percentage values are normalized to MTT metabolism in healthy, untreated HeLa cells and are representative of at least three separate experiments ± standard deviation (*p<0.05, Student’s *t*-test). “Control” refers to non-transfected HeLa cells.

The phototoxicity and subsequent cell death caused by mALAS2-induced PPIX accumulation, when the culture medium was supplemented with glycine, were also investigated in HeLa cells. Cell viability was measured using propidium iodide (data not shown), DAPI staining, and MTT assays (Figure 7) 24 hours post-transfection, 18 hours after glycine addition, and 4 hours after ALA addition. Significant cell death was ascribed to those samples that exhibited more cell death compared to the pIRES2-ZsGreen1-transfected cells with the same glycine concentration as determined by Student’s *t*-tests (p<0.05). Transfection of HeLa cells with the pIRES2-ZsGreen1 plasmid caused a decrease in cell viability by an average of 30%, which can be attributed to the mild cytotoxicity of the transfection reagents [54].

Expressing WT, regardless of the glycine concentration in the culture medium, caused approximately 70% cell death following light exposure, as measured by DAPI staining (Figure 7A–B). In the MTT assays, expression of WT only caused statistically significant cell death (p<0.05) with supplementation of 100 mM glycine, in which case only an average of 15% of cells remained viable (Figure 7C). HeLa cells expressing HPVT or R433K did exhibit a glycine-dependent increase in cell death when the glycine concentration reached 100 mM as measured by both DAPI staining and MTT assays. When 100 mM glycine was added, cells expressing HPVT decreased from an average of 81% viable to 13% viable, and cells expressing R433K decreased from 58% viable to 25% viable as measured by MTT assays (Figure 7C). ALA treatment did not cause significant cell death (p>0.05), regardless of glycine concentration, when measured by either DAPI staining or MTT assays.

## Discussion

Photodynamic therapy (PDT) is a widely utilized clinical procedure for many types of cancer, as well as dermatological conditions such as psoriasis and schleroderma [55]–[57]. PDT is often initiated by application of the pro-drug 5-aminolevulinic acid (ALA) to the patient in order to photosensitize the tissue to be treated [49]. ALA-PDT is very effective, has minimal side effects and is being tested in numerous clinical trials on a wide-spectrum of cancers [49]. There are, however, some limitations to ALA-PDT and other PDTs that provide opportunities for improvement. For example, there is no specific mechanism that targets PPIX to tumor cells after ALA has been applied, other than a slightly preferential uptake of photosensitizers by hyperproliferating cells [58]. Consequently, normal tissue can be damaged from the procedure due to unintentional exposure to light resulting in pain, and conversely, not all hyperproliferating cells necessarily become highly sensitized [55], [59]. In an attempt to restrict photosensitivity by biological, rather than just chemical or physical means, an adenovirus expressing human ALAS2 with mutated HRMs was generated and H1299 lung carcinoma cells were successfully infected [40]. The adenovirus-infected H1299 cells accumulated more PPIX and became more photosensitive than cells supplied with ALA in their media, indicating that delivery of ALAS to tumors could potentially be a useful tool for PDT not only for better targeting, but also for greater photosensitivity [40]. However, the cell death was only 26% even in the presence of deferoxamine [40], leading us to postulate that one or more highly active variants of mALAS2 [31], [35] would increase PPIX accumulation, phototoxicity, and targeted cell death sufficiently to make the approach more clinically attractive.

To explore the ability of mALAS2 variants to cause accumulation of PPIX in mammalian cells, we transfected cells, of both erythroid and non-erythroid lineages, with murine ALAS2-expressing plasmids, and quantified the PPIX fluorescence using FACS analysis. In HeLa cells, we transfected plasmids encoding mALAS2 both with and without mutated presequences and with or without mutations in the mature enzyme sequence (Table 1, Figure 1). As with many mitochondrial proteins, ALAS2 is synthesized in the cytosol and contains a sequence at its N-terminus that targets ALAS2 for mitochondrial import after its synthesis [18]. Within the N-terminus of the ALAS2 precursor, there are three HRMs as recognized by adjacent cysteine-proline residues that have the potential to bind heme. The HRMs located within the presequence of ALAS2 (C11 and C38) have been shown to bind heme [24] and subsequently inhibit the mitochondrial import of ALAS2 [18]. Our experiments support the existing data that the cysteines in the HRMs bind heme, and that mutation of these HRMs relieve the inhibition of mitochondrial import, thus resulting in increased mature, functional ALAS2, as reflected by increased cellular concentrations of PPIX when the HRMs are mutated (Figure 2A). In our study, when the HRMs in the presequences of the mALAS2 constructs were mutated to relieve heme inhibition on mitochondrial import, there were significant increases in PPIX accumulation in HeLa cells expressing WT, HPVT, and R433K (Figure 2A–B).

We tested several mALAS2 variants, covering a range of *in vitro* activity from undetectable to higher than wild-type, for capacity to stimulate PPIX accumulation [31], [35], [36], [60]. Transfection of HeLa cells with the negative control plasmid harboring K313A^Ψ^ resulted in no PPIX increase, as expected (Figure 2A). The mutation of K313 leads to undetectable enzymatic activity *in vitro*, attributable to the role of K313 in formation of a Schiff-base linkage with the PLP cofactor [36], and its additional function as a general base catalyst during the ALAS-catalyzed reaction [3], [28], [36], [61]. In preliminary studies with HeLa cells, it was observed that expression of HPVT^Ψ^, which has seven mutations in its active site loop, yielded significantly more accumulated PPIX than expression of the pIRES2-ZsGreen1 vector control plasmid. HPVT^Ψ^ was chosen for this study as the seven mutations of non-conserved residues in the active site loop resulted in the most active recombinant protein isolated from a variant library at 20°C [35]. Lendrihas *et al*. [35] hypothesized that these mutations in the active site loop, which increase both hydrophilicity and basicity, destabilize the loop by both increasing solubility and eliminating hydrophobic and electrostatic interactions that would typically act to stabilize the loop in its closed confirmation. However, the hyperactivity of HPVT^Ψ^ was temperature-dependent; while at a temperature of 20°C these seven mutations increased the *k*_cat_ value of the recombinant, mature enzyme to more than 10 times of that of the WT enzyme, at 35°C the *k*_cat_ values were nearly the same [35]. While the levels of accumulated PPIX in HeLa cells expressing HPVT were significantly affected by supplementation with glycine (p<0.01), those in K562 cells expressing HPVT were only modestly increased when the medium was supplemented with 100 mM glycine. However, the HPVT-promoted PPIX concentration enrichments were much lower than those in cells expressing WT or R433K (Figures 3 and 4). The relatively low levels of PPIX associated with HPVT expression are presumably due to the temperature-dependent activity profile of this particular mALAS2 variant. HPVT was isolated from a library of mALAS2 variants engineered to possess greater enzymatic activity than wild type mALAS2 by targeting the active site loop to acquire different degrees of mobility [35]. Since the mutations in HPVT destabilized the active site loop and altered the protein conformation [35], it would not be surprising if this variant had a decreased cellular stability. Additionally, the HPVT mutations may affect protein-protein interactions, specifically the ability of mALAS2 to interact with a known binding partner, succinyl-CoA synthetase [62].

The simple addition of the non-toxic substrate glycine to the cell media increased PPIX production by WT, HPVT, and R433K significantly. When the culture medium was supplemented to a final concentration of 10 mM or 100 mM glycine, PPIX production in HeLa cells expressing WT was increased by more than 4- and 6-fold for each concentration, respectively (Figure 3). In K562 cells expressing WT, the effect was even larger, as PPIX increased by more than 5- and 10-fold for 10 mM and 100 mM glycine, respectively (Figure 4A and 4C). The slightly higher increases in PPIX concentrations in the K562 cells when treated with glycine could be due to the stable expression of ALAS2 *versus* the transient transfection used in the HeLa cells. However, the ability of the K562 cells to tolerate higher expression of WT and production of PPIX, in comparison to HeLa cells, might also be attributable to their erythroid lineage. K562 cells are of the erythroleukemic cell type, and bear some proteomic resemblance to undifferentiated erythrocytes [51], [63] and express endogenous ALAS2. Thus while expressing ALAS2 in HeLa cells introduces an enzyme that does not normally exist in epithelial cells, K562 cells may adapt more easily to the expression and up-regulation of the heme biosynthetic pathway. It seems likely that the capacity of mALAS2 to accumulate PPIX will be found to vary in other mammalian cell types as well.

Supplementing the culture medium of K562 cells expressing WT with 100 μM deferoxamine, an iron-chelator previously shown to increase the amount of PPIX in ALAS adenovirus-infected H1299 cells [40], resulted in increase in the mean PPIX fluorescence per cell (Figure 4B and 4D), but it was much less than with glycine supplementation. However, the modest increase was similar to what Gagnebin *et al.* [40] observed in the ALAS adenovirus-infected cells, suggesting the much larger increases reported here with R433K and glycine supplementation represent substantial advancements in our ability to overproduce PPIX in mammalian cells. R433K is only twice as active as WT *in vitro*, and it is reasonable to believe that significantly more active variants could be readily produced via directed evolution, and that these variants would facilitate even greater levels of PPIX accumulation, perhaps to the point of expanding the clinical applicability of PPIX-based PDT.

The HeLa cells that accumulated the highest amount of PPIX were those expressing R433K. The R433K mutation was analyzed in 1998 by Tan *et al*. [31] in a study that identified the nearby R439 as being a conserved residue in many α-family PLP-dependent enzymes, as well as being involved in binding of the amino acid substrate during catalysis [31]. In that study, the kinetic parameters were defined for R439L, R439K, R433L, and R433K. Although not the primary focus of the article, the kinetic parameters for R433L were found to be comparable to those of the wild-type at 37°C, while for R433K the *k*_cat_ was increased by two-fold, with no effect on the Michaelis constants, resulting in a doubling of catalytic efficiency for both substrates [31]. In the results detailed here, expression of R433K in HeLa cells produced a 2- to 3-fold increase in PPIX in comparison to WT, and a 4- to 6-fold increase in PPIX fluorescence in comparison to cells transfected with the pIRES2-ZsGreen1 vector control or HeLa cells alone (Figure 2). When the culture medium was supplemented with glycine, PPIX accumulation increased by 13- to 15-fold in comparison to cells transfected with the pIRES2-ZsGreen1 vector control or HeLa cells alone, and represented the conditions for the highest cellular PPIX accumulation. Given that within these samples there were cells fluorescing with PPIX that were not ZsGreen1-expressing (Figure 3B), some of the PPIX produced in the R433K- and WT-expressing HeLa cells appears to have been transported out of the cells into the culture medium, where it was then taken up by non-transfected cells. These populations of red-fluorescing cells were comparable to those cells in culture medium supplemented with ALA (Figure 3B). This is a very important finding in terms of the potential of these constructs for photodynamic therapy, as it indicates that the PPIX produced within a delivery cell could be transported into surrounding diseased tissue and accumulate to clinically relevant levels.

The subcellular distribution of a photosensitizing agent might have important consequences in regards to PDT efficacy [64]. Fluorescence microscopy was successfully utilized to visualize PPIX accumulation in R433K expressing HeLa cells. Due to the extremely short time (a few seconds) it took for PPIX to photobleach, the resolution could not be optimized for visibility of individual organelles such as mitochondria and nuclei. However, it was possible to observe that PPIX had accumulated in the plasma membranes of the R433K expressing cells; and this fluorescence was not seen in the pIRES2-ZsGreen1 vector-expressing cells (Figure 5). It also appears that PPIX can actively exit the transfected cells, potentially via ABC transporters, and enter surrounding cells. Presumably, the lipophilic PPIX accumulated in the organelle membranes as well, but direct microscopic confirmation of PPIX accrual in organelle membranes awaits approaches resulting in better resolution than we were able to achieve here.

In order to study the photosensitivity of cells that accumulated PPIX (caused by expression of WT, HPVT, and R433K), we performed light treatments using an incandescent lamp, which emits light in the visible and infrared spectral regions (400–1000 nm). The cells were exposed for 10 minutes before FACS analysis of PPIX and cell death. Expression of the ZsGreen1 protein alone caused a toxicity that was independent of light treatment, and thus significant cell death was measured against the pIRES2-ZsGreen1 vector-transfected cells (p<0.05) as opposed to HeLa cells alone (Figure 7). Regardless of glycine concentration, the expression of WT led to a significant increase in cell death by 1.4- to 2-fold. R433K and HPVT, when the culture medium was supplemented with 100 mM glycine, also caused significant increases in cell death by 2.1- and 1.6-fold, respectively. With no or 10 mM glycine supplementation the error was too high for any of the increases to be significant, although there was a trend towards increased cell death. For WT and R433K, increased PPIX accumulation correlates with increased cell death after light treatment. In spite of the more modest increases in PPIX accumulation, light-induced death also occurred in HPVT-expressing cells. This finding led us to suggest that toxicity inherent to the overproduced HPVT protein is the other contributing factor to cell death. Although purified recombinant, mature HPVT is more active than WT at 37°C (with an enhancement of approximately 1.5 in the *k*_cat_ value at 37°C *vs.* a 15.5-fold increased *k*_cat_ at 20°C) [65], when expressed in mammalian cells, HPVT is not as active as WT, as determined by PPIX fluorescence. Since expression of HPVT paired with glycine supplementation caused significant cell death (Figure 7), the potential role of another toxic heme pathway intermediate, such as the pro-oxidant ALA [66]–[69], in causing cell death, is plausible. However, at the present, different levels of protein expression [Quantification of ALAS2 and variant protein levels was not feasible due to the unavailability of a functional ALAS2 antibody.] and distinct modulation of the enzymatic activity between ALAS2 and variants cannot be ruled out as possibilities. Treatment with 100 μM ALA, a positive control for PPIX accumulation, caused a similar cell death after light exposure to expression of the ZsGreen1 protein alone. In summary, the highest cell death was seen in light-treated HeLa cells expressing ZsGreen1 and either WT, HPVT, or R433K, in culture medium supplemented with 100 mM glycine. The total cell death was observed to be as high as 90% in the cells expressing both ZsGreen1 and R433K. This represents a substantial improvement upon the 26% cell death reported previously [40], and indicates that this approach, if carefully developed, may eventually find some clinical utility.

Because ALAS2, especially highly stable and active variants of ALAS2, would be useful in the development of multiplex cancer treatments involving PDT, we experimented with combination PDT and drug treatments of HeLa cells. Paclitaxel (Taxol) is currently approved in the United States for the treatment of AIDS-related Kaposi sarcoma [70], breast cancer [71], non-small lung cell lung cancer [72], and ovarian cancer [73]. Paclitaxel induces apoptosis in cancer cells by binding to tubulin and inhibiting the disassembly of microtubules, thereby resulting in the inhibition of cell division [46], [74]. As expected, paclitaxel did not affect PPIX accumulation, as indicated by the similar mean PPIX fluorescence values between untreated and paclitaxel-treated cells (Figure 2B). However, paclitaxel had an additive effect to PDT and increased cell death in all samples by 10–25%. HeLa cells expressing R433K treated with paclitaxel exhibited the highest percentage of cell death (Figure 6A–B). Ever since PDT was first shown to be able to elicit an immune response [75], many recent advances in PDT are aiming toward creating PDT-generated cancer vaccines [76]. Using highly active and stable ALAS2 variants as part of a vaccine strategy for photosensitization may be useful for this vaccine approach. Further experiments, both in cell culture and live animals, are necessary to test for potential immune response stimulated by ALAS2-PDT.

In this study, we have shown that transfecting mammalian cells, of both erythroid and non-erythroid lineages, with mALAS2 variants, is an effective way to stimulate cellular PPIX accumulation. Furthermore, supplementing the culture medium with glycine vastly increases the intracellular PPIX levels when cells express either WT or R433K. These data offer new ways to accumulate high levels of the photosensitizer PPIX in cancer cells for more targetable and efficient PDT. Human ALAS2 variants with higher than normal activity are now known to occur in nature, and are associated with a form of erythropoietic protoporphyria known as X-linked dominant protoporphyria, which is characterized by a 24-fold increase in erythrocyte PPIX concentrations [39]. It would be appropriate if these variants were eventually utilized in PDT, and could thereby allow clinicians to exploit one disease to treat or cure others.

## Supporting Information

Figure S1 **Porphyrin accumulation in HeLa cells through expression of mALAS2 compared to supplementation of culture medium with ALA.** HeLa cells transfected with mALAS2 variants were analyzed 24 hours after transfection, and ALA-treated HeLa cells were analyzed 4 hours after addition of ALA to the culture medium. Mean PPIX fluorescence values were compared to those of the pIRES2-ZsGreen1-transfected cells control; each of PPIX data sets is representative of three separate experiments ± standard deviation [a.u., arbitrary units].
(TIF)
