# Supplementary figures and images for: Expression of Murine 5-Aminolevulinate Synthase Variants Causes Protoporphyrin IX Accumulation and Light-Induced Mammalian Cell Death
Source: PLoS One. 2014 Apr 9;9(4):e93078. doi: 10.1371/journal.pone.0093078 (PMC3981678; doi:10.1371/journal.pone.0093078)

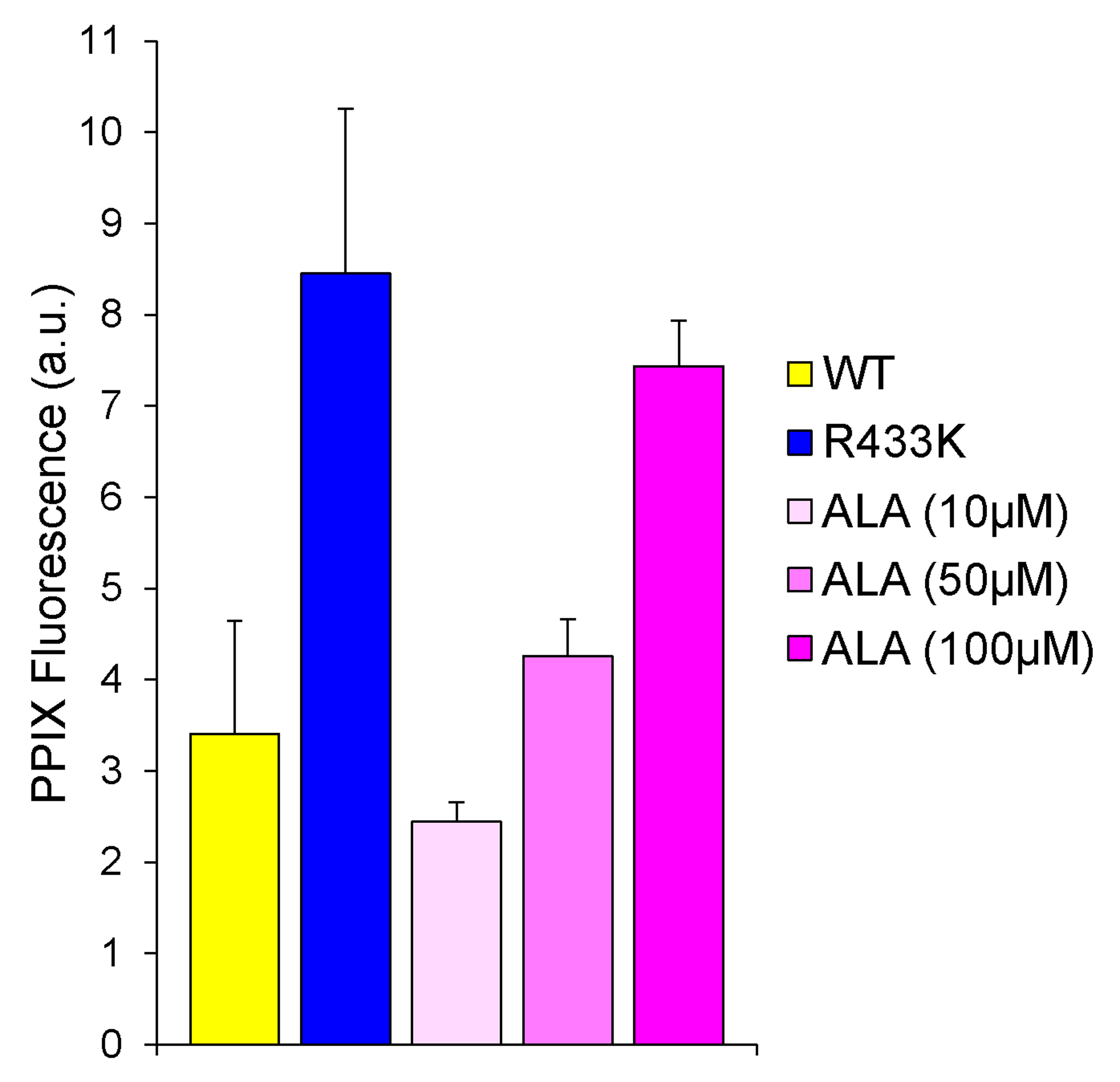

Supplement: Figure S1 — Porphyrin accumulation in HeLa cells through expression of mALAS2 compared to supplementation of culture medium with ALA. HeLa cells transfected with mALAS2 variants were analyzed 24 hours after transfection, and ALA-treated HeLa cells were analyzed 4 hours after addition of ALA to the culture medium. Mean PPIX fluorescence values were compared to those of the pIRES2-ZsGreen1-transfected cells control; each of PPIX data sets is representative of three separate experiments ± standard deviation [a.u., arbitrary units]. (TIF) [file pone.0093078.s001.tif]
